# Dietary flexibility of Bale monkeys (*Chlorocebus djamdjamensis*) in southern Ethiopia: effects of habitat degradation and life in fragments

**DOI:** 10.1186/s12898-018-0161-4

**Published:** 2018-02-06

**Authors:** Addisu Mekonnen, Peter J. Fashing, Afework Bekele, R. Adriana Hernandez-Aguilar, Eli K. Rueness, Nils Chr. Stenseth

**Affiliations:** 1 Centre for Ecological and Evolutionary Synthesis (CEES), Department of Biosciences, University of Oslo, P.O. Box 1066, Blindern, 0316 Oslo, Norway; 2 Department of Zoological Sciences, Addis Ababa University, P.O. Box: 1176, Addis Ababa, Ethiopia; 3 Department of Anthropology and Environmental Studies Program, California State University Fullerton, Fullerton, CA 92834 USA

**Keywords:** Bamboo, Continuous forest, Feeding ecology, Fragmented forest, Human-wildlife conflict, Specialist folivore

## Abstract

**Background:**

Understanding the effects of habitat modification on the feeding strategies of threatened species is essential to designing effective conservation management plans. Bale monkeys (*Chlorocebus djamdjamensis*) are endemic to the rapidly shrinking montane forests of the southern Ethiopian Highlands. Most populations inhabit continuous bamboo forest subsisting largely on the young leaves and shoots of a single species of bamboo. Because of habitat disturbance in recent decades, however, there are now also several dozen small populations inhabiting isolated forest fragments where bamboo has been degraded. During 12-months, we assessed Bale monkey responses to habitat degradation by comparing habitat composition, phenological patterns, and feeding ecology in a largely undisturbed continuous forest (Continuous groups A and B) and in two fragments (Patchy and Hilltop groups).

**Results:**

We found that habitat quality and food availability were much lower in fragments than in continuous forest. In response to the relative scarcity of bamboo in fragments, Bale monkeys spent significantly less time feeding on the young leaves and shoots of bamboo and significantly more time feeding on non-bamboo young leaves, fruits, seeds, stems, petioles, and insects in fragments than in continuous forest. Groups in fragments also broadened their diets to incorporate many more plant species (Patchy: ≥ 47 and Hilltop: ≥ 35 species)—including several forbs, graminoids and cultivated crops—than groups in continuous forest (Continuous A: 12 and Continuous B: 8 species). Nevertheless, bamboo was still the top food species for Patchy group (30% of diet) as well as for both continuous forest groups (mean = 81%). However, in Hilltop group, for which bamboo was especially scarce, *Bothriochloa radicans* (Poaceae), a grass, was the top dietary species (15% of diet) and bamboo ranked 10th (2%).

**Conclusions:**

We demonstrate that Bale monkeys are more dietarily flexible than previously thought and able to cope with some degradation of their primary bamboo forest habitat. However, crop raiding and other terrestrial foraging habits more common among fragment groups may place them at greater risk of hunting by humans. Thus, longitudinal monitoring is necessary to evaluate the long-term viability of Bale monkey populations in fragmented habitats.

**Electronic supplementary material:**

The online version of this article (10.1186/s12898-018-0161-4) contains supplementary material, which is available to authorized users.

## Background

Habitat loss and degradation by humans are the major threats to biodiversity worldwide [1, 2]. Widespread disturbance to formerly intact forests, particularly in the tropics, is resulting in increasing fragmentation of habitats and biological populations [3]. Given that the global human population is expected to continue to increase in the coming decades, resulting habitat alterations may cause the extinction of thousands of species, including many mammals [4–6]. Habitat degradation modifies vegetation composition and structure, consequently reducing habitat quality and food availability for species inhabiting an area [6–10]. This decrease in food availability, in turn, lowers the carrying capacity of populations, and, in extreme cases, results in extirpation or extinction [7, 8, 11].

Currently, many populations are restricted to small isolated forest patches surrounded by human-dominated landscapes [12–14]. The persistence of these populations, therefore, depends on their ability to cope with change and the minimum size and quality of fragments required to sustain them [15–17]. One of the central challenges that must be overcome by populations in fragments is meeting their dietary needs in habitats in which the diversity and abundance of plant species has been substantially altered [7, 11, 18].

Among mammals, specialist species are declining across the world and are at higher risk of extinction or extirpation than generalist species [19]. Specialist folivores are particularly threatened [20] because they tend to be forest-dwelling, arboreal, and/or sensitive to changes in forest structure [14, 21–24]. Examples include marsupials like koalas (*Phascolarctos cinereus*) and greater gliders (*Petauroides volans*) that feed primarily on *Eucalyptus* [23], giant pandas (*Ailuropoda melanoleuca*) and red pandas (*Ailurus fulgens*) that feed almost exclusively on bamboo [14, 21] and primates like bamboo lemurs (*Hapalemur* spp., *Prolemur simus*) and golden monkeys (*Cercopithecus mitis kandti*) that feed mostly on bamboo [25, 26]. Bamboo specialist mammals, in particular, often have special morphological, anatomical, behavioural and ecological adaptations to cope with diets rich in cellulose and toxic plant secondary metabolites (PSMs), including cyanide [27, 28]. Food choice in mammalian folivores is influenced by multiple factors, including the availability of specific food items or species within their habitat (e.g., [29, 30]), and the energy, protein, fiber and toxic PSM concentrations in foods [31, 32]. While dietary specialists, including some specialist folivores, are generally associated with narrow ecological tolerances [24, 33, 34] some taxa exhibit enough ecological flexibility to cope with habitat degradation [35–37].

Although habitat degradation is increasingly common in tropical forests [38], intensive studies comparing the feeding ecology within species of populations in continuous versus fragmented forests are lacking for most mammals, including most specialist folivores. However, a handful of such studies have been carried out on tropical primates. Dietary responses to degradation and life in fragments among primates are varied, though common strategies include increasing consumption of (1) abundant fallback foods like leaves (*Alouatta palliata*: [39, 40], *Ateles geoffroyi*: [41], *Propithecus diadema*: [42]), (2) foods from secondary growth species, including lianas and climbers (*Ateles geoffroyi*: [41], *Alouatta palliata*: [43]) or graminoids and forbs (*Hapalemur griseus*: [44], *H. meridionalis*: [45]), or (3) human crops and exotic species: (*Alouatta guariba clamitans*: [46], *Macaca sylvanus*: [47]). Furthermore, some primate taxa persist in forest fragments by increasing the plant species richness of their diet (*Alouatta pigra*: [48], *Cercopithecus mitis boutourlinii*: [49]) while others cope by eating a less species rich diet (*Propithecus diadema*: [42], *Ateles geoffroyi*: [41]). In some cases, fragments are too small or primates lack the ecological plasticity to survive on the foods present, resulting in widespread local extirpation of populations from their former habitats (*Trachypithecus pileatus*, *Macaca assamensis* and *Hoolock hoolock*: [50]).

Understanding the dietary responses of individual species to habitat degradation and life in fragments is therefore crucial to designing and implementing appropriate species-based management strategies [51, 52], especially for dietary specialists which are expected to be less flexible at coping with degradation of their habitats than generalist species [24, 53]. For example, until now, no research has yet been conducted to assess the effects of habitat degradation and life in fragments on the feeding strategies of the Bale monkey (*Chlorocebus djamdjamensis*), an arboreal dietary specialist endemic to the montane forests of the southern Ethiopian Highlands. The Bale monkey is unusual among primates and other mammals for its intense specialization on a single species of bamboo (*Arundinaria alpina*), which accounts for 77% of its diet in continuous forest [54, 55]. The Bale monkey is thought to be at high risk of extirpation because of its specialized niche, small geographic distribution, and the ongoing deforestation occurring across much of its range [54, 56–58]. As a result, the species is currently classified as Vulnerable by the International Union for Conservation of Nature (IUCN) [56].

In its high degree of specialization, the Bale monkey appears to provide a striking contrast to its five sister species: vervet monkeys (*Chlorocebus pygerythrus*), grivet monkeys (*C. aethiops*), green monkeys (*C. sabaeus*), Malbrouck monkeys (*C. cynosuros*) and tantalus monkeys (*C. tantalus*). Two of these sister species, vervets and grivets, are also native—though not endemic—to Ethiopia and are parapatric to Bale monkeys [59, 60]. All members of the genus *Chlorocebus*, except Bale monkeys, are terrestrial generalists that consume varied omnivorous diets and inhabit a wide range of savanna woodland and grassland habitats over large geographic ranges in equatorial or southern Africa [61–63]. Incidentally, an analogous situation exists among monkeys in the genus *Cercopithecus* where one taxon, the golden monkey (*Cercopithecus mitis kandti*), is a bamboo specialist while other taxa, including other* C. mitis* subspecies, tend to be dietary and habitat generalists [63, 64].

Intriguingly, the recent discovery of Bale monkey populations during surveys in a few dozen heavily-degraded forest fragments, some with little bamboo left [57], suggested the species might be of greater ecological flexibility than previously believed [54–56, 65]. This unexpected discovery created the need to evaluate the strategies the monkeys employ in response to habitat degradation and life in fragments by comparing groups inhabiting fragmented habitats with those in continuous forest. We therefore undertook a study comparing the activity, ranging, and dietary patterns of Bale monkeys in fragmented and continuous forests. We recently published evidence that Bale monkeys in fragmented habitats adopt an *energy minimization strategy*—moving less, feeding less, resting more, and traveling over shorter distances per hour than conspecifics in continuous forest [66]. Along with examining energetic responses to degradation, we sought to determine the dietary strategies Bale monkeys use to cope with the relative scarcity of bamboo in fragments.

The specific aims of the study described here were thus to assess the effects of habitat degradation and life in fragments on (1) habitat quality and temporal patterns of food availability and (2) Bale monkey dietary composition, diversity and selectivity by comparing the feeding ecology between populations in continuous and fragmented forests. We also sought to (3) compare the patterns of dietary flexibility exhibited by Bale monkeys in our study with those of their five sister *Chlorocebus* species [63], as well as with those of other bamboo-eating mammals, including several other primates (e.g., *Cercopithecus mitis kandti* [67], *Macaca assamensis* [68], *Prolemur simus* [26], *Hapalemur* spp. [26]) and red and giant pandas [14, 34, 69]. We hypothesized that any reduction in habitat quality in forest fragments would strongly influence the feeding strategies of Bale monkeys. In particular, we predicted that the anticipated lower abundance of bamboo in fragments [57] would lead Bale monkeys there to consume a greater diversity of food items, plant species and growth forms, including human foods on nearby farms, than conspecifics in continuous forest. We also predicted that Bale monkeys in continuous forest would be bamboo specialists [54], but that conspecifics in fragments would exploit diets more similar to those of other more generalized *Chlorocebus* species [61, 70].

## Methods

### Study site and habitat characteristics

We carried out our study in the continuous Odobullu Forest (06°50′–6°56′N and 40°06′–40°12′E) and two forest fragments (6°44′–06°45′N and 38°48′–38°51′E) in the southern Ethiopian Highlands [66]. Odobullu Forest (hereafter continuous forest) is a large forest within which bamboo is abundant. It covers 141 km^2^ (14,100 ha) at elevations ranging from 1500 m to 3300 m asl and lies east of Bale Mountains National Park [54]. The continuous forest consists of four habitat types: mostly bamboo forest and tree-dominated forest but also shrubland and occasional grasslands [55]. It is partially protected by a privately-owned hunting company, Ethiopian Rift Valley Safaris, and disturbance in the home range of our study groups is uncommon due to the steep terrain and remoteness of the area.

Kokosa forest fragment (hereafter Patchy fragment) consists of degraded bamboo with large trees set amidst a matrix of human settlement, cultivated land, shrubland and grazing land. It covers an area of 162 ha and ranges in elevation from 2534 m to 2780 m asl. Most of Patchy fragment is privately owned by local people, though a portion is owned by the community collectively [66]. Selective logging of bamboo is common today.

Afursa forest fragment (hereafter Hilltop fragment) is set upon a hilltop and consists of a mix of secondary forest, shrubland, and *Eucalyptus* plantation with graminoid and forb cover underneath. Bamboo has been nearly extirpated. Hilltop fragment covers an area of 34 ha at elevations ranging from 2582 m to 2790 m asl and is surrounded by an anthropogenic matrix of cultivated lands, pastures and human settlements. Currently, the district government forbids cutting of trees and use of the fragment for grazing. The edge of the fragment, especially the *Eucalyptus* plantation, is still used illegally for grazing. Both the Patchy and Hilltop fragments were dominated by bamboo forest only three decades ago [57]. The distance between Hilltop and Patchy fragments is 9 km and they have been separated from one another by human settlement, grazing land and agriculture for many decades [57]. The forest fragments are separated from the continuous forest by ~ 160 km [66].

### Study groups

We selected four Bale monkey groups for this study: two groups within the continuous bamboo forest (hereafter Continuous A and Continuous B) with overlapping home ranges (29% overlap for Continuous A; 47% overlap for Continuous B) [66], one group in the Patchy fragment (Patchy group) and one group in the Hilltop fragment (Hilltop group). The home ranges of continuous forest groups (Continuous A and Continuous B) consisted of exclusively bamboo forest (53.7 and 55.6%) and mixed bamboo forest habitats (46.3 and 44.4%). In contrast, the home ranges of fragment groups consisted of more variable habitat types. Patchy group’s range consisted of five habitat classes: grazing land (37.9%), shrubland (29.5%), mixed bamboo forest (17.1%), tree-dominated forest (8.0%) and cultivated land (7.5%) while Hilltop group’s range consisted of four habitat classes: shrubland (50.4%), tree-dominated forest (22.7%), *Eucalyptus* plantation (24.3%) and grazing land (2.7%) [66]. A.M. and two assistants habituated these groups to human observers for 4 months from March to June 2013 by following each group from dawn to dusk on a near daily basis. We identified 10–15 members of each focal group by their distinctive natural markings (e.g., coat color, facial features, tail shape). Group sizes were: Continuous A, 65 individuals; Continuous B, 38 individuals; Patchy, 28 individuals; and Hilltop, 23 individuals [66].

### Climate

We recorded climatic data at the continuous forest (Fly campsite, elevation 2758 m asl; 1.5–2.0 km from the two study groups) and at Patchy fragment (Kokosa campsite, elevation 2634 m asl; 1.5 km from Patchy fragment). We measured daily rainfall using Oregon wireless rain gauges and recorded the daily maximum and minimum temperatures using Taylor digital waterproof maximum/minimum thermometers. We assumed that the rainfall and temperature patterns are similar in each of the two fragments because they are both small, located only 9 km apart, occur at similar elevations, and are oriented in the same north–south and east–west directions. We calculated the monthly and annual rainfall for the period July 2013 to June 2014. We also used the daily maximum and minimum temperatures to calculate monthly means for these variables and calculated annual means by taking the averages of the monthly means.

**Fig. 1:**
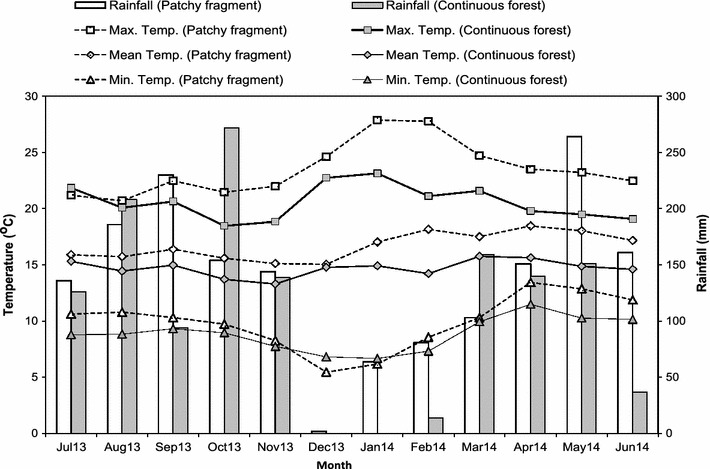
Monthly temperature and rainfall patterns in continuous forest and one forest fragment. Monthly temperature (mean, mean minimum and mean maximum) and rainfall patterns at Odobullu continuous forest (2758 m asl) and Kokosa (Patchy) forest fragment (2634 m asl) from July 2013 to June 2014

Though annual rainfall was higher in the fragments (1676 mm SE ± 20.6) than in the continuous forest (1340 mm SE ± 24.8), this difference was not significant (ANOVA: df = 1; F = 2.31; P = 0.136) (Fig. 1). Both study areas were characterized by bimodal rainfall with a long wet season and a short dry season (Fig. 1) but rainfall was less strongly seasonal in the forest fragments than in the continuous forest (Fig. 1). Mean annual temperature (16.7 °C SE ± 0.4) was significantly higher in the forest fragments than in the continuous forest (14.7 °C SE ± 0.2) (ANOVA: df = 1; F = 48.71; P < 0.001).

### Vegetation description and temporal patterns of food availability

To examine whether the diet of Bale monkeys was influenced by resource availability, we sampled the vegetation in the ranges of our study groups using two complementary techniques. First, we enumerated all large trees with diameter at breast height (DBH) ≥ 10 cm in 12–24, 50 m × 10 m vegetation quadrats along randomly selected vegetation transects in the home range of each study group. Within quadrats, we measured and recorded the species name, growth form and DBH (in cm) for each tree. Second, we also randomly selected 50% of the vegetation quadrats in each group’s range within which we counted and identified all plants ≥ 2 m tall to species level. This second vegetation enumeration technique enabled us to sample bamboo, shrubs and forbs that the monkeys depend on but that are < 10 cm DBH. For the bamboo sampled with this second technique, we also recorded the DBH of each culm.

In each group’s home range, we calculated the stem density for all plant species ≥ 2 m tall and basal area (cm^2^/ha) for all large tree species (DBH ≥ 10 cm) and bamboo. We assessed the degree of stem density overlap between the home ranges of the study groups using the Morisita–Horn similarity index, which takes into account both relative abundance and species richness [71]. We classified plant growth forms into five categories: bamboo, trees, shrubs, lianas (including climbers and epiphytes), and forbs. To estimate the biomass of each large tree species and bamboo, we calculated the basal area (BA) of each tree species from the DBH recorded using the formula (BA = [0.5 × DBH]^2^ × *π*) [72].

To evaluate temporal changes in the availability of potential food resources, we carried out monthly phenological assessments over an annual cycle for selected food plant species found at each of the study sites (see [66] for additional details). These species were selected for monitoring because they had been food species for Bale monkeys in a previous 8-month study in continuous forest at Odobullu [54]. At the start of our study, we marked and identified 10–15 individuals of these food species which included: trees (DBH ≥ 10 cm), bamboo (*A. alpina*), and shrubs. We assigned every monitored plant a relative abundance score for each of its potential food items (young leaves, mature leaves, flowers, ripe fruits, and shoots) via visual inspection, using binoculars where necessary. Relative abundance score ranged from 0 (item absent from plant) to 8 (plant fully laden with the item) at intervals of 1 [66].

We analyzed phenological data from eight species: five trees (*Canthium oligocarpum*, *Dombeya torrida*, *Galiniera saxifraga*, *Hagenia abyssinica*, and *Ilex mitis*), two shrubs (*Rubus apetalus* and *Bothriocline schimperi*) and bamboo (*A. alpina*). Ultimately, these species cumulatively accounted for 92.6% of the diet of Continuous A; 93.4% for Continuous B, 50.9% for Patchy and 44.5% for Hilltop groups. The lower contribution of monitored plants to the diets of fragment groups resulted from these groups consuming much less bamboo as well as a greater variety of food species, including difficult-to-monitor insects, graminoids and forbs (cf., [73]), than continuous forest groups. We calculated the monthly mean phenological scores for young leaves, fruits, flowers, and shoots for each individual plant species. We calculated the monthly food availability index (FAI) for each plant part by multiplying the mean phenology scores of species *i* with the mean basal area of species *i* and density of the corresponding species *i* per ha [72].

### Feeding ecology

We collected activity data from July 2013 to June 2014 using instantaneous scan sampling [74] at 15-min intervals for up to 5 min duration, typically from 0700 to 1730 [66]. During scans, when a monkey was observed feeding, we recorded the type of food item, growth form and species. We recorded food items as bamboo young leaves, bamboo mature leaves, non-bamboo young leaves (from all species other than bamboo), non-bamboo mature leaves, bamboo shoots, bamboo branchlets (young and thin stems emerging from branches), roots, flowers, fruits, seeds, stems, petioles, insects or mushrooms. We recorded plant growth form as tree, bamboo, shrub, liana (including climbers and epiphytes), forb, or graminoid (grass or sedge). Although most food species consumed were identified in the field, species that could not be identified were collected for taxonomic identification at the National Herbarium in Addis Ababa. We recorded a food item as insects when the monkey was observed manipulating tree bark, searching through dead leaves or directly consuming insects [54]. We collected 28,583 individual records (hereafter records) during 2085 h of observation (Continuous A = 441; Continuous B = 432; Patchy fragment = 601; Hilltop fragment = 611) over the 12-month study period [66]. Feeding accounted for 15,302 of these records: Continuous A, 3027 records (monthly mean ± SD records = 252.3 ± 58.8); Continuous B, 3086 records (257.2, ± 72.2); Patchy fragment, 5239 records (436.6 ± 61.5); and Hilltop fragment, 3950 records (329.2 ± 68.1). Feeding accounted for 54.9% of Continuous A’s, 56.2% of Continuous B’s, 51.5% of Patchy’s and 53.2% of Hilltop’s overall activity budget [66]. Monthly sampling effort was evenly distributed among groups throughout the year.

We assessed dietary composition for each month by determining the proportion of different food items, growth forms and species consumed in each study group. We then calculated annual consumption of food items, growth forms and species as the means of the 12 monthly values for each category. We combined four food items (mature leaves, branchlets, roots and mushrooms) into the category “other” in our analyses because each individually accounted for < 1% of the overall percentage of feeding records. We also compared the identity and contributions of the top five plant species in the diets of each group. We calculated the relative dietary preference (i.e., *food selection ratios*) by dividing the proportion of annual percentage of feeding records on a particular species *i* by the percentage stem density of species *i* in the study group’s home range. A *selected* food species is consumed more frequently than expected based on its proportional representation in the group’s home range [72]. A food selection ratio of 1 indicates no selectivity for that food plant species, < 1 indicates a food species is avoided and > 1 indicates a food species is selected. We were only able to calculate selection ratios for trees, bamboo, shrubs, and lianas because stem density cannot be evaluated using the same methods for graminoids and forbs.

To estimate the annual plant species richness of the diet for each study group, we pooled the data from all sampling months within each group. We calculated within-month and annual dietary diversity indices for each group using the Shannon–Wiener index (*H*′), dominance index (*D*) and evenness index (*J*) [71] using the software PAST [75]. To assess differences in inter-month dietary similarity among groups in continuous forest and forest fragments, we calculated the inter-month Morisita–Horn’s similarity indices (C_H_) of each group [76] using EstimateS [77]. To assess the annual diet overlap among groups in continuous forest and forest fragments, we also calculated between group Morisita–Horn similarity indices. The index (C_H_) ranges from 0 (no diet overlap) to 1 (complete diet overlap).

### Statistical analyses

We conducted all statistical tests using R version 3.3.2 [78] with significance level set at P ≤ 0.05 unless otherwise stated. We tested data for normality and homogeneity of variances using the Shapiro–Wilk and Levene tests, respectively. We initially calculated and compared variables for each study group individually and examined the differences using the one-way analysis of variance (ANOVA) model followed by the Tukey honest significant difference (HSD) post hoc test. When the results for both groups within continuous forest and fragments were similar, we combined these groups for data analysis unless otherwise stated.

The completeness of plant species recorded in the diet is dependent on sample size. Therefore, we constructed a sample-based rarefaction curve plotting species richness with sampling effort (number of observation days) using PAleontological STatistics (PAST) software [75] to perform a valid comparison of dietary species richness among groups. To examine differences in monthly Shannon–Wiener dietary diversity indices among groups in continuous forest and forest fragments, we conducted a one-way analysis of variance (ANOVA) using the log transformed monthly values as replicas. To examine differences in monthly dietary dominance and evenness indices between continuous forest and fragment groups, we used a generalized linear model (GLM) with a quasibinomial error distribution and logit link-function as recommended for proportional data [79]. We also used a GLM with a quasibinomial error distribution and logit link-function to test for differences in between-month Morisita–Horn similarity indices among groups. We identified differences among groups by post hoc multiple comparisons using function ‘glht’ from R package multcomp [80]. We used a one-way ANOVA to test for differences in the percentage consumption of each food item and growth form between continuous forest and fragment groups. We applied logit transformations of proportion data before statistical analysis to normalize the data as recommended by Warton and Hui [81]. We used linear regressions to assess whether the availability of non-bamboo young leaves, bamboo young leaves, fruits, flowers, and bamboo shoots was a good predictor of their consumption in each study group.

## Results

### Vegetation description and temporal variation in resource availability

The vegetation in the ranges of Bale monkey groups inhabiting forest fragments was more diverse (55 species) than in the ranges of groups in continuous forest (23 species) (Additional file 1). We found 24 tree, 14 shrub, 11 liana, 4 forb, 1 bamboo, and 1 fern species in the home ranges of fragment groups but only 12 tree, 2 shrub, 7 liana, 1 forb and 1 bamboo species in the ranges of continuous forest groups (Additional file 1). The ranges of the two continuous forest groups were much more similar in plant species composition and abundance (19 of 23 species shared, Morisita–Horn similarity index = 0.99) than the ranges of the two fragment groups (28 of 55 species shared, Morisita–Horn similarity index = 0.40).

**Fig. 2:**
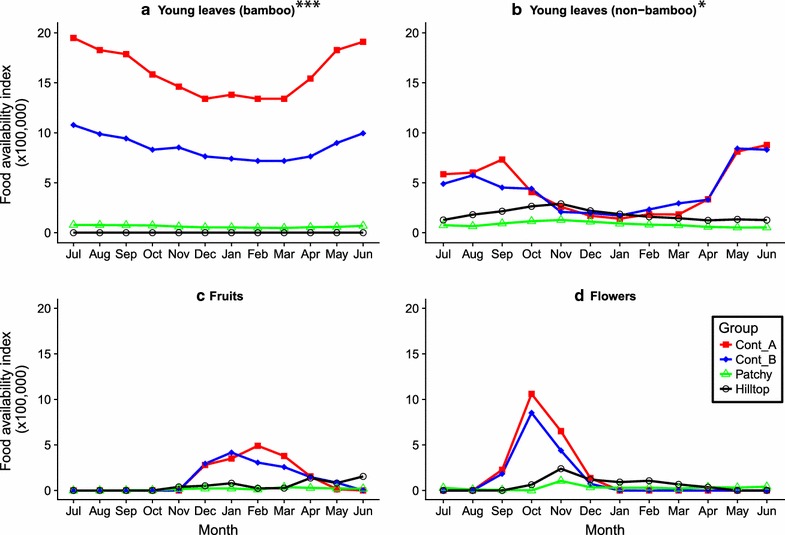
Differences in the monthly availability indices (units/ha) of major food items between Bale monkey groups in continuous forest (Continuous A, [Cont_A], Continuous B, [Cont_B]) and forest fragments (Patchy and Hilltop)

Bale monkey foods were much more abundant in continuous forest than in fragments. Monthly food availability indices of bamboo young leaves (ANOVA: F = 544.00, df = 1, P < 0.001), non-bamboo young leaves (ANOVA: F = 17.17, df = 1, P < 0.001), and fruits (ANOVA: F = 4.19, df = 1, P = 0.05) were all significantly higher in continuous forest than in forest fragments (Fig. 2). Bamboo young leaves were abundant throughout the year in continuous forest, consistently available at low levels in Patchy fragment, and consistently scarce in Hilltop fragment. However, there was no difference in the availability indices of flowers (ANOVA: F = 1.44, df = 1, P = 0.243) and bamboo shoots (ANOVA: F = 0.88, df = 1, P = 0.357) between continuous forest and fragment groups.

### Dietary species richness, diversity and similarity

Overall, at least 65 plant species (1 bamboo, 12 trees, 5 shrubs, 8 lianas, ≥ 25 forbs and ≥ 14 graminoids) were food sources for Bale monkeys. They also ate one species of mushroom and presumably many unidentified species of insects. Dietary species richness was much higher in groups inhabiting forest fragments (≥ 61 species: Patchy ≥ 47 species; Hilltop ≥ 35 species) than in groups inhabiting continuous forests (12 species: Continuous A = 12 species; Continuous B = 8 species). The rarefaction curves for dietary plant species richness reached a plateau for each of the four study groups, suggesting we sampled intensively enough to obtain robust values for dietary species richness in all groups (Additional file 2).

**Fig. 3:**
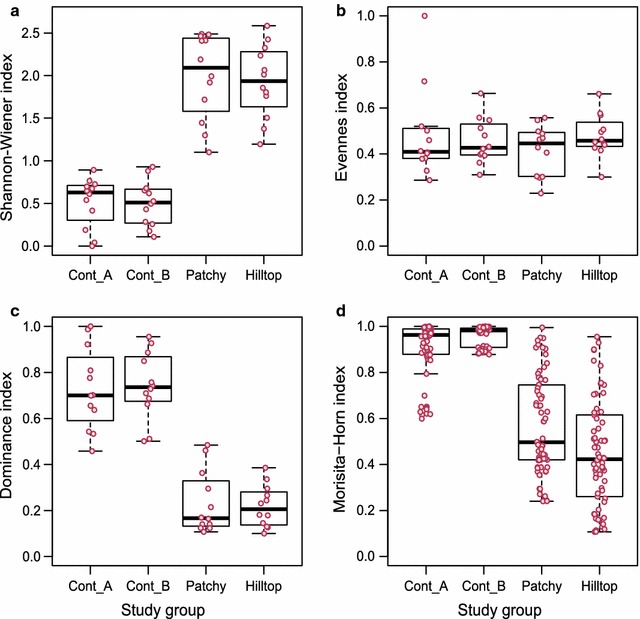
Box plots showing dietary diversity, evenness, dominance and similarity indices among groups in continuous forest and fragments. Box plots show variations among groups in continuous forest (Continuous A, [Cont_A], Continuous B, [Cont_B]) and forest fragments (Patchy and Hilltop) in **a** Shannon–Wiener dietary diversity index, *H*′, **b** dietary plant species evenness index, **c** dietary plant species dominance index, *D* and **d** between-month dietary plant species similarity index. Dots represent the corresponding data set in each study group, the line in the box indicates the median of the corresponding index value, and the box shows the 25 and 75% interquartile. Vertical dotted lines represent the acceptable range with IQD (interquartile distance) multiplied by 1.5. All groups showed significant differences (P < 0.01)

The mean monthly Shannon–Wiener diversity index (*H*′) of food species was significantly higher in fragments than in continuous forest (ANOVA: F = 178.60, df = 1, P < 0.001; Fig. 3a). However, mean monthly dietary species evenness (*J*) was not significantly different between groups inhabiting fragments and those in continuous forest (GLM: F = 0.35, df = 1, P = 0.555; Fig. 3b). Lastly, mean monthly food plant species dominance was significantly higher for groups inhabiting continuous forest than for those in fragments (GLM: F = 163.60, df = 1, P < 0.001; Fig. 3c). Between-month dietary species similarity was significantly greater for groups in continuous forest than for groups in forest fragments (GLM: F = 380.80, df = 1, P < 0.001; Fig. 3d). Annual dietary species overlap was much lower between the two fragment groups (21 of 61 species shared; Morisita–Horn similarity index = 0.36) than for the groups in continuous forest (8 of 12 species shared; Morisita–Horn similarity index = 0.99).

### Food item consumption

**Fig. 4:**
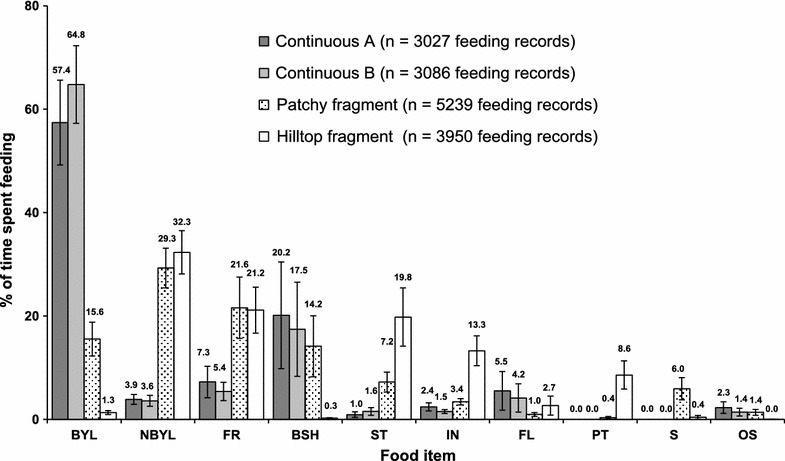
The proportion of feeding records devoted to different food items by the four Bale monkey groups. N = 12 months, mean ± SE: *BYL* bamboo young leaves, *NBYL* non-bamboo young leaves, *BSH* bamboo shoots, *FL* flowers, *FR* fruit, *ST* stems, *PT* petioles, *S* seeds, *IN* insects, *OS* others

Groups in continuous forest spent significantly more time feeding on bamboo young leaves (61.1% vs. 8.5%; ANOVA: F = 54.19; P < 0.001), and significantly less time feeding on non-bamboo young leaves (3.8% vs. 30.8%; ANOVA: F = 44.66; P < 0.001), fruits (6.4% vs. 21.4%; ANOVA: F = 19.66; P = 0.001), stems (1.3% vs. 13.5%; ANOVA: F = 31.15; P < 0.001), petioles (0.0% vs. 4.5%; ANOVA: F = 20.00; P < 0.001), seeds (0.0% vs. 3.2%; ANOVA: F = 10.95; P = 0.002), and insects (2.0% vs. 8.4%; ANOVA: F = 10.45; P = 0.002) than groups in forest fragments (Fig. 4). Most of the difference in insect consumption between continuous forest and fragment groups was driven by Hilltop group (13.7%; Patchy: 3.3%; Continuous A: 2.4%; Continuous B: 1.5%). There was no difference in the consumption of bamboo shoots (18.8% vs. 7.2%; ANOVA: F = 0.001; P = 0.975), flowers (4.9% vs. 1.9%; ANOVA: F = 0.01; P = 0.941), and ‘other’ items (1.7% vs. 0.7%; ANOVA: F = 0.25; P = 0.619) between continuous forest and fragment groups.

### Consumption of different growth forms

In forest fragments, a total of 10 tree, 1 bamboo, 5 shrub, 7 liana, 24 forb, 14 graminoid and 1 mushroom species were food sources for Bale monkeys whereas in continuous forest only 3 tree, 1 bamboo, 1 shrub, 4 liana, 2 forb, and 1 graminoid species were food sources for the monkeys. Groups in fragments spent less time feeding on bamboo (15.9% vs. 81.2%; ANOVA: F = 68.77, P < 0.001) and more time feeding on trees (22.7% vs. 11.8%; ANOVA: F = 3.30, P = 0.029), shrubs (12.7% vs. 0.1%; ANOVA: F = 337.10, P < 0.001), forbs (21.0% vs. 0.1%; ANOVA: F = 345.20, P < 0.001), and graminoids (17.1% vs. 0.7%; ANOVA: F = 98.33, P < 0.001) than groups in continuous forest (Additional file 3). There was no significant difference in the consumption of lianas (2.1% vs. 4.1%; ANOVA: F = 1.06, P = 0.309) between continuous forest and fragment groups (Additional file 3).

### Top five species consumption

The cumulative percentage of the annual diet accounted for by the top five plant species was much higher in groups inhabiting continuous forest (continuous A = 96.2%; Continuous B = 97.3%) than in groups in fragments (Patchy = 62.0%; Hilltop = 50.4%). Bamboo (*Arundinaria alpina*) was the top food species consumed in both continuous forest groups (Mean = 81.2%) and in Patchy fragment group (30.2%) but was only the 10th most eaten food species in Hilltop fragment group (1.6%). Instead, in Hilltop fragment where bamboo was especially rare, a grass, *Bothriochloa radicans*, was the top plant species (15.3%) in the annual diet (Table 1). *Bothriochloa radicans* was only a minor (< 1%) dietary species for the other study groups, though 5 other graminoid species were more commonly consumed than *B. radicans* by the group in Patchy fragment. *Galiniera saxifraga*, a tree, was the second most frequent food source in continuous forest (Mean = 6.6%) and Hilltop fragment (11.8%) and the third most frequent food source in Patchy fragment group (7.4%).

**Table 1:** Annual percentage of feeding records on different food items from each plant species among the four Bale monkey groups

Family	Percentage of feeding records for each food item												
	Species consumed	Growth form	BYL	NBYL	SH	FL	FR	ST	PT	S	IN	OS	Total
Continuous A													
Poaceae	*Arundinaria alpina*	Bamboo	57.69	–	19.66	–	–	–	–	–	–	2.02	79.37
Rubiaceae	*Galiniera saxifraga*	Tree	–	0.15	–	–	7.38	–	–	–	–	–	7.53
Sterculiaceae	*Dombeya torrida*	Tree	–	–	–	5.96	–	–	–	–	–	–	5.96
Asteraceae	*Mikaniopsis clematoides*	Liana	–	2.37	–	–	–	–	–	–	–	0.06	2.43
Urticaceae	*Urera hypselodendron*	Liana	–	0.03	–	–	–	0.85	–	–	–	–	0.89
Poaceae	*Bothriochloa radicans*	Graminoid	–	0.72	–	–	–	–	–	–	–	0.06	0.78
Vitaceae	*Cypostemma adenocaule*	Liana	–	0.20	–	–	–	–	–	–	–	–	0.20
Rubiaceae	*Galium spurium*	Forb	–	0.16	–	–	–	–	–	–	–	–	0.16
Rosaceae	*Rubus apetalus*	Shrub	–	0.03	–	–	0.06	0.03	–	–	–	–	0.12
Asclepiadaceae	*Oxystelma bornouense*	Liana	–	0.06	–	–	–	–	–	–	–	–	0.06
Sapindaceae	*Allophylus macrobotrys*	Tree	–	–	–	–	0.06	–	–	–	–	–	0.06
Acanthaceae	*Acanthopale pubescens*	Forb	–	0.03	–	–	–	–	–	–	–	–	0.03
	Insects		–	–	–	–	–	–	–	–	2.42	–	2.42
Total			57.69	3.76	19.66	5.96	7.50	0.88	–	–	2.42	2.14	100.00
Continuous B													
Poaceae	*Arundinaria alpina*	Bamboo	64.20	–	17.59	–	–	–	–	–	–	1.26	83.05
Rubiaceae	*Galiniera saxifraga*	Tree	–	0.19	–	0.03	5.36	–	–	–	–	–	5.58
Sterculiaceae	*Dombeya torrida*	Tree	–	–	–	4.51	–	–	–	–	–	–	4.51
Asteraceae	*Mikaniopsis clematoides*	Liana	–	2.16	–	–	–	–	–	–	–	–	2.16
Urticaceae	*Urera hypselodendron*	Liana	–	0.26	–	–	–	1.74	–	–	–	–	2.00
Poaceae	*Bothriochloa radicans*	Graminoid	–	0.63	–	–	–	–	–	–	–	0.05	0.68
Vitaceae	*Cypostemma adenocaule*	Liana	–	0.43	–	–	–	–	–	–	–	–	0.43
Acanthaceae	*Acanthopale pubescens*	Forb	–	0.08	–	–	–	–	–	–	–	–	0.08
	Insects		–	–	–	–	–	–	–	–	1.50	–	1.50
Total			64.20	3.76	17.59	4.54	5.36	1.74	–	–	1.50	1.31	100.00
Patchy fragment													
Poaceae	*Arundinaria alpina*	Bamboo	15.70	–	13.34	–	–	–	–	–	–	1.14	30.18
Apiaceae	*Centellia asiatica*	Forb	–	12.76	–	–	–	–	–	–	–	–	12.76
Rubiaceae	*Galiniera saxifraga*	Tree	–	0.05	–	–	7.35	–	–	–	–	–	7.39
Rosaceae	*Rubus apetalus*	Shrub	–	0.04	–	–	6.08	0.82	–	–	–	–	6.94
Poaceae	*Cynodon dactylon*	Graminoid	–	3.85	–	–	–	0.86	–	–	–	–	4.71
Fabaceae	*Trifolium tembense*	Forb	–	4.16	–	–	–	–	–	–	–	–	4.16
Rubiaceae	*Canthium oligocarpum*	Tree	–	0.17	–	0.23	3.49	–	–	–	–	0.03	3.91
Poaceae	*Hordeum vulgare* ^a^	Graminoid	–	0.17	–	–	–	–	–	3.44	–	–	3.61
Poaceae	*Poa annua*	Graminoid	–	0.96	–	–	–	–	–	2.28	–	–	3.25
Myrsinaceae	*Maesa lanceolata*	Tree	–	–	–	–	2.74	0.36	–	–	–	–	3.10
Aquifo liaceae	*Ilex mitis*	Tree	–	0.48	–	0.43	0.22	1.02	–	–	–	–	2.15
Musaceae	*Ensete ventricosum* ^a^	Forb	–	0.09	1.00	–	–	0.87	–	–	–	–	1.96
Papilionaceae	*Erythrina brucei*	Tree	–	0.32	–	–	–	0.68	0.10	–	–	0.02	1.11
Poaceae	*Zea mays* ^a^	Graminoid	–	–	–	–	1.05	0.05	–	–	–	–	1.09
Apiaceae	*Agrocharis melanantha*	Forb	–	0.98	–	–	–	–	–	–	–	–	0.98
Asteraceae	*Carduus schimperi*	Forb	–	–	–	0.33	0.02	0.51	–	–	–	–	0.85
Urticaceae	*Urera hypselodendron*	Liana	–	–	–	–	0.19	0.59	–	–	–	–	0.78
Asteraceae	*Bothriocline schimperi*	Shrub	–	–	–	–	–	0.62	–	–	–	–	0.62
Araceae	*Arisaema schimperianum*	Forb	–	0.12	–	–	–	0.40	–	–	–	–	0.52
Poaceae	*Pennisetum thunbergii*	Graminoid	–	0.40	–	–	–	–	–	–	–	–	0.40
Poaceae	*Bothriochloa radicans*	Graminoid	–	0.37	–	–	–	–	–	–	–	–	0.37
Caryophyllaceae	*Drymaria cordata*	Forb	–	0.33	–	–	–	0.02	–	–	–	–	0.35
Amaryllidaceae	*Allium* sp.	Graminoid	–	0.22	–	–	–	–	–	–	–	0.05	0.27
Polygonaceae	*Rumex nepalensis*	Forb	–	0.26	–	–	–	–	–	–	–	–	0.26
Fabaceae	*Trifolium substerraneum*	Forb	–	0.23	–	–	–	–	–	–	–	–	0.23
Solanaceae	*Solanum tuberosum* ^a^	Forb	–	0.20	–	–	0.02	–	–	–	–	–	0.22
Poaceae	*Deschampsia caespitosa*	Graminoid	–	–	–	–	–	–	–	0.21	–	–	0.21
Rosaceae	*Hagenia abyssinica*	Tree	–	–	–	–	–	–	0.19	–	–	–	0.19
Oleaceae	*Jasminum abyssinicum*	Liana	–	0.08	–	–	0.10	–	–	–	–	–	0.18
Casuarinaceae	*Casuarina equisetifolia*	Tree	–	–	–	–	–	–	–	0.15	–	–	0.15
Myrsinaceae	*Embelia schimperi*	Liana	–	0.03	–	–	0.09	0.02	–	–	–	–	0.14
Cucurbitaceae	*Lagenaria abyssinica*	Liana	–	–	–	–	0.14	–	–	–	–	–	0.14
Balsaminaceae	*Impatiens hochstetteri*	Forb	–	–	–	–	–	0.12	–	–	–	–	0.12
Poaceae	*Avena fatua*	Graminoid	–	0.11	–	–	–	–	–	–	–	–	0.11
Rubiaceae	*Galium spurium*	Forb	–	0.10	–	–	–	–	–	–	–	–	0.10
Cupressaceae	*Juniperus procera*	Tree	–	–	–	–	0.09	–	–	–	–	–	0.09
Agaricaceae	*Mushroom*	Fungi	–	–	–	–	–	–	–	–	–	0.09	0.09
Poaceae	*Snowdenia polystacha*	Graminoid	–	0.09	–	–	–	–	–	–	–	–	0.09
Asclepiadaceae	*Oxystelma bornouense*	Liana	–	0.08	–	–	–	–	–	–	–	–	0.08
Asteraceae	*Vernonia sp.*	Shrub	–	–	–	–	–	0.06	–	–	–	–	0.06
Compositae	*Lactuca glandulifera*	Liana	–	0.06	–	–	–	–	–	–	–	–	0.06
Lamiaceae	*Pycnostachys eminii*	Shrub	–	–	–	–	–	0.06	–	–	–	–	0.06
Poaceae	*Cyperus rigidifolius*	Graminoid	–	–	–	–	–	0.05	–	–	–	–	0.05
Asteraceae	*Carduus leptacanthus*	Forb	–	–	–	–	–	0.04	–	–	–	–	0.04
Poaceae	*Eleusine floccifolia*	Graminoid	–	0.04	–	–	–	–	–	–	–	–	0.04
Lamiaceae	*Plectranthus alpinus*	Forb	–	–	–	0.02	–	–	–	–	–	–	0.02
Asphodelaceae	*Kniphofia* sp.	Forb	–	0.02	–	–	–	–	–	–	–	–	0.02
Lamiaceae	*Plectranthus garckeanus*	Forb	–	–	–	–	–	0.02	–	–	–	–	0.02
	Unidentified Grass	Graminoid	–	1.63	–	–	–	–	–	–	–	–	1.63
	Unidentified Herb	Forb	–	0.85	–	–	–	–	–	–	–	–	0.85
	Insects		–	–	–	–	–	–	–	–	3.34	–	3.34
Total			15.70	29.19	14.34	1.00	21.58	7.15	0.29	6.08	3.34	1.32	100.00
Hilltop fragment													
Poaceae	*Bothriochloa radicans*	Graminoid	–	15.27	–	–	–	–	–	–	–	–	15.27
Rubiaceae	*Galiniera saxifraga*	Tree	–	–	–	–	11.73	0.05	–	–	–	–	11.77
Rosaceae	*Rubus apetalus*	Shrub	–	–	–	–	7.47	1.95	–	–	–	–	9.41
Rosaceae	*Hagenia abyssinica*	Tree	–	–	–	–	–	–	8.44	–	–	–	8.44
Asteraceae	*Bothriocline schimperi*	Shrub	–	–	–	–	–	8.06	–	–	–	–	8.06
Apiaceae	*Centellia asiatica*	Forb	–	7.92	–	–	–	–	–	–	–	–	7.92
Aquifoliaceae	*Ilex mitis*	Tree	–	–	–	2.28	–	3.51	–	–	–	–	5.79
Apiaceae	*Haplosciadium abyssinicum*	Forb	–	3.52	–	–	–	–	–	–	–	–	3.52
Urticaceae	*Urera hypselodendron*	Liana	–	–	–	–	0.71	1.69	–	–	–	–	2.40
Poaceae	*Arundinaria alpina*	Bamboo	1.34	–	0.27	–	–	–	–	–	–	–	1.61
Commelinaceae	*Commelina sp.*	Forb	–	0.31	–	–	–	1.11	–	–	–	–	1.42
Fabaceae	*Trifolium tembense*	Forb	–	1.08	–	–	–	–	–	–	–	–	1.08
Asteraceae	*Crassocephalum macropappus*	Forb	–	0.79	–	–	–	0.21	–	–	–	–	1.00
Cupressaceae	*Juniperus procera*	Tree	–	–	–	–	1.00	–	–	–	–	–	1.00
Lamiaceae	*Plectranthus alpinus*	Forb	–	0.22	–	0.07	–	0.59	–	–	–	–	0.88
Poaceae	*Poa annua*	Graminoid	–	0.19	–	–	–	–	–	0.50	–	–	0.69
Urticaceae	*Pilea rivularis*	Forb	–	0.17	–	–	–	0.33	–	–	–	–	0.50
Poaceae	*Cynodon dactylon*	Graminoid	–	0.49	–	–	–	–	–	–	–	–	0.49
Caryophyllaceae	*Drymaria cordata*	Forb	–	0.29	–	–	–	0.11	–	–	–	–	0.39
Balsaminaceae	*Impatiens hochstetteri*	Forb	–	–	–	0.04	–	0.34	–	–	–	–	0.38
Asteraceae	*Mikaniopsis clematoides*	Liana	–	0.11	–	–	–	0.22	–	–	–	–	0.33
	*Keshansho*	Graminoid	–	0.33	–	–	–	–	–	–	–	–	0.33
Asteraceae	*Carduus schimperi*	Forb	–	–	–	0.11	–	0.09	–	–	–	–	0.20
Asteraceae	*Vernonia rueppellii*	Shrub	–	0.02	–	–	–	0.11	–	–	–	–	0.13
Solanaceae	*Discopodium penninervium*	Tree	–	0.04	–	0.09	–	–	–	–	–	–	0.13
Papilionaceae	*Erythrina brucei*	Tree	–	–	–	–	–	0.03	0.10	–	–	–	0.12
Rosaceae	*Alchemilla fischeri*	Forb	–	0.12	–	–	–	–	–	–	–	–	0.12
Poaceae	*Zea mays* ^a^	Graminoid	–	–	–	–	0.11	–	–	–	–	–	0.11
Olaeaceae	*Jasminum abyssinicum*	Liana	–	0.11	–	–	–	–	–	–	–	–	0.11
Asteraceae	*Echinops sp.*	Forb	–	–	–	–	–	0.09	–	–	–	–	0.09
Urticaceae	*Girardinia bullosa*	Forb	–	0.09	–	–	–	–	–	–	–	–	0.09
Capparaceae	*Ritchiea albersii*	Tree	–	–	–	–	–	–	0.05	–	–	–	0.05
Urticaceae	*Urtica simensis*	Forb	–	0.04	–	–	–	–	–	–	–	–	0.04
Agaricaceae	*Agaricaceae* sp.	Fungi	–	–	–	–	–	–	–	–	–	0.03	0.03
Crassulaceae	*Crassula alsinoides*	Forb	–	0.02	–	–	–	–	–	–	–	–	0.02
	Sheshako	Shrub	–	–	–	–	–	0.02	–	–	–	–	0.02
	Unidentified Grass	Graminoid	–	1.46	–	–	–	–	–	–	–	–	1.46
	Unidentified Herb	Forb	–	0.88	–	–	–	–	–	–	–	–	0.88
	Insects		–	–	–	–	–	–	–	–	13.68	–	13.68
Total			1.34	33.49	0.27	2.59	21.02	18.50	8.59	0.50	13.68	0.03	100.00

^a^Cultivated food species

### Dietary preference

The selection ratios of bamboo, tree, shrub, and liana food species accounting for > 0.5% of the annual diets of the study groups are presented in Table 2. Despite its dominance in the diets of the continuous forest groups, bamboo (*Arundinaria alpina*) had selection ratios of just below 1.00 in continuous forest (Continuous A = 0.94 and Continuous B = 0.95) owing to its extremely high stem density in this forest type. Although they ate much less bamboo, the fragment groups also exhibited comparable selection ratios to those of the continuous groups for bamboo (Patchy = 0.76; Hilltop: 1.00). The most selected plant species by both continuous forest groups was the tree *Dombeya torrida* with selection ratios of 6.78 (Continuous A) and 12.19 (Continuous B), respectively. For the fragment groups, the most selected food species were the trees *Erythrina brucei* (27.83) in Patchy fragment and *Hagenia abyssinica* (10.42) in Hilltop fragment. However, it should be noted that the top food species in the diet of Hilltop group was a graminoid species, *B. radicans*, for which a selection ratio could not be calculated. The one species that exhibited consistently high selection ratios and ranked among the top three species for dietary selectivity across groups was the tree *Galiniera saxifraga* (Continuous A: 2.20, 2nd rank; Continuous B: 1.85, 3rd rank; Patchy: 3.73, 2nd rank; Hilltop: 2.68, 3rd rank) from which Bale monkeys ate primarily fruits.

**Table 2:** Selection ratios of food species contributing ≥ 0.5% to the diet of the four Bale monkey groups

Group	Species	Growth form	% of diet^a^	% of stem density	Selection ratio (rank)
Continuous A	*Arundinaria alpina*	Bamboo	79.37	84.74	0.94 (3)
	*Galiniera saxifraga*	Tree	7.53	3.42	2.20 (2)
	*Dombeya torrida*	Tree	5.96	0.88	6.78 (1)
	*Mikaniopsis clematoides*	Liana	2.43	3.37	0.72 (4)
	*Urera hypselodendron*	Liana	0.89	1.83	0.48 (5)
Continuous B	*Arundinaria alpina*	Bamboo	83.05	87.12	0.95 (5)
	*Galiniera saxifraga*	Tree	5.58	3.02	1.85 (3)
	*Dombeya torrida*	Tree	4.51	0.37	12.19 (1)
	*Mikaniopsis clematoides*	Liana	2.16	0.49	4.37 (2)
	*Urera hypselodendron*	Liana	2.00	2.00	1.00 (4)
Patchy fragment	*Arundinaria alpina*	Bamboo	30.18	39.59	0.76 (5)
	*Galiniera saxifraga*	Tree	7.39	1.98	3.73 (2)
	*Rubus apetalus*	Shrub	6.94	15.26	0.45 (8)
	*Canthium oligocarpum*	Tree	3.91	1.48	2.64 (4)
	*Maesa lanceolata*	Tree	3.10	4.52	0.69 (6)
	*Ilex mitis*	Tree	2.15	0.64	3.36 (3)
	*Erythrina brucei*	Tree	1.11	0.04	27.83 (1)
	*Urera hypselodendron*	Liana	0.78	1.39	0.56 (7)
	*Bothriocline schimperi*	Shrub	0.62	8.35	0.07 (9)
Hilltop fragment	*Galiniera saxifraga*	Tree	11.77	4.39	2.68 (3)
	*Rubus apetalus*	Shrub	9.41	19.46	0.48 (8)
	*Hagenia abyssinica*	Tree	8.44	0.81	10.42 (1)
	*Bothriocline schimperi*	Shrub	8.06	15.01	0.54 (7)
	*Ilex mitis*	Tree	5.79	4.71	1.23 (4)
	*Urera hypselodendron*	Liana	2.40	3.27	0.73 (6)
	*Arundinaria alpina*	Bamboo	1.61	1.61	1.00 (5)
	*Juniperus procera*	Tree	1.00	0.16	6.25 (2)

Selection ratios of tree, bamboo, shrub, and liana are calculated for each group based on percentage of stem density accounted for by the plant species in continuous forest (Continuous A and Continuous B) and forest fragments (Patchy and Hilltop)

^a^Rank ordered based on annual diet of plant species used for selection ratio. We were unable to calculate dietary preference for forbs and graminoids because their abundance could not be determined in the same manner as for the other plant growth forms

### Temporal variability in food item availability and consumption

Bamboo young leaf and shoot consumption were significantly correlated with availability over time in Continuous groups A and B and in Patchy fragment group (Table 3). It is possible that similar relationships between these variables also existed in Hilltop fragment, but we did not track changes in bamboo abundance over time here because of the low density and small sizes of individuals of bamboo at this site. The consumption of fruits and flowers were also significantly correlated with availability for both groups inhabiting continuous forest and fruit consumption was significantly correlated with availability for Hilltop fragment group (Table 3).

**Table 3:** Linear regressions between food availability index and percentage consumption of plant food items among the four Bale monkey groups

Food item	Continuous	R^2^_adj_	P value	Fragments	R^2^_adj_	P value
Bamboo young leaves	Continuous A	0.26	*0.052*	Patchy	0.50	*0.006*
	Continuous B	0.52	*0.005*	Hilltop	–	–
Non-bamboo young leaves	Continuous A	0.09	0.180	Patchy	0.07	0.204
	Continuous B	0.12	0.145	Hilltop	− 0.07	0.634
Fruit	Continuous A	0.87	*0.005*	Patchy	0.25	0.060
	Continuous B	0.85	*<* *0.001*	Hilltop	0.55	*0.004*
Flower	Continuous A	0.64	*0.023*	Patchy	0.10	0.981
	Continuous B	0.60	*0.002*	Hilltop	0.14	0.124
Bamboo shoots	Continuous A	0.86	*<* *0.001*	Patchy	0.54	*0.004*
	Continuous B	0.92	*<* *0.001*	Hilltop	–	–

Bale monkey groups in continuous forest (Continuous A, Continuous B) and forest fragments (Patchy and Hilltop) (N = 12 months) (*P* value in italic indicates significant correlations)

## Discussion

### Dietary responses to habitat degradation by Bale monkeys compared to other primates

Habitat degradation affects plant species richness, diversity and structure in forest fragments, ultimately reducing the availability of food resources for many primate species [48, 82, 83]. Specifically, the destruction or degradation of mature continuous forest promotes the growth in light gaps of pioneer species including fast-growing graminoids, forbs, shrubs, lianas and trees [9, 44, 84–86]. In our study, Bale monkeys in fragments exploited many of these pioneer species (Table 1), broadening their diet to include a far greater diversity of plant species (indigenous, exotic, and/or cultivated) and growth forms than conspecifics in continuous forest.

Primates inhabiting fragments frequently eat a higher percentage of leaves than conspecifics in continuous forest [41, 42, 46, 49]. Bale monkeys, however, ate a much lower percentage of leaves in fragments than in continuous forest largely because of the lower availability of bamboo in the former. In fragments, Bale monkeys modified their diet by increasing consumption of fruits, stems, petioles and insects as well as the leaves of a number of species other than bamboo. Interestingly, the much higher fruit consumption in fragments occurred despite fruit being significantly less available in fragments than in continuous forest.

Another common dietary response to habitat degradation among primates is to consume more secondary successional species, including shrubs, forbs, or graminoids [39, 41–44, 87]. The Bale monkeys in our study clearly fit this pattern, obtaining more than half their diet from shrubs, forbs, and graminoids in forest fragments (Additional file 3).

Primates in fragments also exhibit a tendency to consume exotic species and/or human crops from surrounding human matrix [46, 47, 88], a habitat absent from the ranges of conspecifics in continuous forest. Bale monkeys in both fragments in our study engaged in crop-raiding, though the group in Patchy fragment, whose range included more areas of human use [66], had a diet containing a higher overall percentage of crops. Farmer responses to crop raiding by Bale monkeys included throwing stones, hunting with spears, chasing them with dogs, or positioning scarecrows in cultivated areas (Mekonnen, personal observation). In addition to crops, Bale monkeys in fragments also consumed bamboo planted near the homes of local people, triggering additional human-monkey conflict, particularly at Patchy fragment (Mekonnen, personal observation).

Lastly, the species richness of primate diets in fragments often differs from in continuous forests, increasing substantially for some primates (e.g., *Alouatta pigra* [48]; *Cercopithecus mitis boutourlinii* [49]), while decreasing for others (e.g., *Ateles geoffroyi* [41]; *Propithecus diadema* [42]). Bale monkeys appear to adopt the former approach, consuming many more plant—and probably insect—species in fragments. The strategy of continuous forest Bale monkeys to focus primarily on bamboo is simply not an option for monkeys in fragments where bamboo populations have been degraded or almost eradicated and the monkeys must diversify their diet to survive.

### Dietary flexibility in Bale monkeys relative to other *Chlorocebus* species

Several of the *Chlorocebus* species are well-studied and eat varied diets with the top food item ranging from fruit in Nigerian (*C. tantalus*: [89]) and Senegalese (*C. sabaeus*: [90]) populations to gum or flowers in Kenyan populations (*C. pygerythrus*: [70, 91, 92]) (Table 4). Among *Chlorocebus*, Bale monkeys (*C. djamdjamensis*) are unique in their heavy reliance on the young leaves and shoots of bamboo in relatively undisturbed continuous forest habitats.

**Table 4:** Percentage of feeding time devoted to different food items by wild populations of *Chlorocebus*

Species	Study length (mon)	Group (n or name)	BYL	OYL	ML	BSH	GRB	TL	FR	S	TF	FL	ST	GU	AP	OS	No. spp	Site, Country	Reference
*Chlorocebus djamdjamensis*	12	CA	57.7	3.1	0.5	19.7	0.7	81.7	7.5	0.0	7.5	6.0	0.9	0.0	2.4	1.6	12	Odobullu (CF), Ethiopia	This study
*C. djamdjamensis*	12	CB	64.2	3.2	0.4	17.6	0.6	86.0	5.4	0.0	5.4	4.5	1.7	0.0	1.5	0.9	8	Odobullu (CF), Ethiopia	This study
*C. djamdjamensis*	12	Patchy	15.7	21.4	0.0	14.3	7.8	59.2	21.6	6.1	27.2	1.0	7.2	0.0	3.3	1.6	47	Kokosa (FF), Ethiopia	This study
*C. djamdjamensis*	12	Hilltop	1.3	15.8	0.1	0.3	17.7	35.2	21.0	0.5	21.5	2.6	18.5	0.0	13.7	8.5^a^	35	Afursa (FF), Ethiopia	This study
*C. djamdjamensis*	8	2	73.0	7.2	1.1	1.5	0.9	82.8	9.6	0.0	9.6	3.1	1.4	0.0	2.3	0.9	11	Odobullu (CF), Ethiopia	Mekonnen et al. [54]
*C. pygerythrus*	11	2	0.0	0.8	–	0.0	4.7	5.5	7.0	10.2	17.2	7.6	8.0	47.9	0.7	13.1	–	Laikipia (*Acacia xanthophloea* Woodland), Kenya	Isbell et al. [61]
*C. pygerythrus*	11	2	0.0	3.2	–	0.0	3.4	6.6	1.7	6.6	8.3	2.3	0.0	39.5	7.5	35.8	–	Laikipia (*A. drepanolobium* Woodland), Kenya	Isbell et al. [61]
*C. pygerythrus*	9	3	0.0	–	–	0.0	–	26.6	11.1	2.6	13.7	0.0	0.0	30.0	7.7	0.2	–	Amboseli, Kenya	Wrangham and Waterman [91]
*C. pygerythrus*	26	1	0.0	–	–	0.0	8.3	8.3	5.8	19.6	25.4	44.7	0.0	0.0	0.0	1.3	–	Saumburu-Isiolo, Kenya	Whitten [70]
*C. sabaeus*			0.0	–	–	0.0	0.0	–	50.2	12.8	63.0	13.0	0.0	0.0	13.1	10.9	–	Mt. Assirik, Senegal	Harrison [90]
*C. tantalus*	11	1	0.0	–	–	0.0	0.0	20.5	49.2	0.0	49.2	5.3	0.0	0.0	25.1	0.0	28	Ngel Nyaki, Nigeria	Agmen et al. [89]

*BYL* bamboo young leaves, *OYL* young leaves except bamboo and grass, *ML* mature leaves, *BSH* bamboo shoots, *GRL* grass blades, *TL* total leaves, *FR* fruits, *S* seeds, *TF* total fruits, *FL* flowers, *ST* stems, *PT* petioles, *GU* gum, *AP* animal prey, *OS* others

Habitat: *CF* continuous forest, *FF* fragmented forest

^a^8.3 petiole

Intriguingly, our study revealed that *C. djamdjamensis* inhabiting fragments consumed diets more comparable to those of the other less specialized *Chlorocebus* species than to continuous forest-dwelling *C. djamdjamensis* populations. For example, percentages of fruit and graminoid consumption by *C. djamdjamensis* in fragments were similar to those reported for East African *C. pygerythrus* populations (Table 4). Further, levels of invertebrate consumption by the Hilltop group of *C. djamdjamensis* mirrored levels of invertebrate consumption by *C. sabaeus* in West Africa (Table 4). Lastly, *C. tantalus*’s diet in West Africa was 2–3 times more species rich than the diets of *C. djamdjamensis* in continuous forest though actually somewhat less species rich than the diets of *C. djamdjamensis* in fragments (Table 4). Though the one dietary commonality among *C. djamdjamensis* groups in our study was a greater reliance on leaves than in any of the other *Chlorocebus* spp. (maximum 25% of the diet), consumption of leaves still varied widely among *C. djamdjamensis* groups.

The remarkable dietary flexibility exhibited by *C. djamdjamensis* in fragments has at least two possible explanations. First, they may retain some of the ancestral ecological flexibility characteristic of other members of the genus *Chlorocebus*, only expressing this plasticity when habitat degradation requires them to diversify their diets beyond primarily bamboo and a handful of other species. A second possibility is that genetic introgression (hybridization) between *C. djamdjamensis* and parapatric *C. aethiops* and *C. pygerythrus* in fragmented forest areas [57, 60, 93] endows some *C. djamdjamensis* populations with the ability to radically alter their diets in fragments.

### Bamboo consumption across bamboo eating mammals

Adaptation to bamboo-dominated forests and diets appears to have evolved at least six times among the mammals: giant pandas in China [34, 94], red pandas in India, Nepal, Bhutan, Myanmar, and China [69], bamboo lemurs (*Hapalemur*/*Prolemur* spp.) in Madagascar [26, 95], Assamese macaques (*Macaca assamensis*) in China [68, 96], golden monkeys in Uganda and Rwanda [67, 97], and Bale monkeys in Ethiopia (this study; Table 5). Most of the primate taxa are members of ecologically-flexible genera (*Macaca*: [98]; *Chlorocebus*: [63]) or species (*Cercopithecus mitis*: [64, 99]), while giant and red pandas belong to different more specialized families in the order *Carnivora* [69]. Among the other bamboo-eating primates, the closest phylogenetically and geographically to *Chlorocebus djamdjamensis* is *Cercopithecus mitis kandti* (Table 5). Both taxa feed primarily on a single species of African highland bamboo (*Arundinaria alpina*) though *C. mitis kandti* rely on it less than *C. djamdjamensis* populations in continuous forest and more than *C. djamdjamensis* populations in fragmented forest ([54, 100]; This study).

**Table 5:** Percentage of feeding time devoted to different food items and bamboo species by Bale monkeys, bamboo lemurs and other bamboo-eating primates

Species	Study length (month)	YL	ML	SH	GRB	TL	ST	FL	FR	S	TF	AP	OS	Bamboo	No. of species	Habitat	Site, Country	Reference
*Chlorocebus djamdjamensis* (CA)	12	60.7	0,5	19.7	0.7	81.7	0.9	6.0	7.5	0.0	7.5	2.4	1.6	79.4^b^	12	Montane bamboo forest, CF	Odobullu, Ethiopia	This study
*C. djamdjamensis* (CB)	12	67.3	0.4	17.6	0.6	86.0	1.7	4.5	5.4	0.0	5.4	1.5	0.9	83.1^b^	8	Montane bamboo forest, CF	Odobullu, Ethiopia	This study
*C. djamdjamensis* (PF)	12	37.7	0.0	14.3	7.8	59.2	7.2	1.0	21.6	6.1	27.2	3.3	1.6	30.2^b^	47	Fragmented forest, FF	Kokosa, Ethiopia	This study
*C. djamdjamensis* (HF)	12	17.1	0.1	0.3	17.7	35.2	18.5	2.6	21.0	0.5	21.5	13.7	8.63	1.6^b^	35	Fragmented montane forest FF	Afursa, Ethiopia	This study
*C. djamdjamensis*	8	79.3	1.1	1.5	0.9	82.8	1.4	3.1	9.6	–	9.6	2.3	0.9	76.7^b^	11	Montane bamboo forest, CF	Odobullu, Ethiopia	Mekonnen et al. [54]
*Cercopithecus mitis kandti* ^a^	8	44.0	0.1	–	0.0	44.1	3.4	14.8	22.5	–	22.5	14.3	1.1	52.4^b^	16	Montane bamboo forest	Mgahinga, Uganda	Twinomugisha et al. [100]
*Hapalemur aureus*	24	–	–	–	0.0	91	–	–	4	–	4	–	5	78^c^	≥21	Submontane rain forest	Ranomafana, Madagascar	Tan [26]
*H. griseus*	13	0.3	6.3	89.1	0.0	95.7	–	0.4	1.2	–	1.2	–	2.7	89.1^c^	12	Domain forest	Ranomafana, Madagascar	Overdorff et al. [108]
*H. griseus*	24	–	–	–	–	92	–	–	5	–	5	–	3	72^c^	≥ 24	Submontane rain forest	Ranomafana, Madagascar	Tan [26]
*H. meridionalis* (n = 3)	12	8.8	0.0	0.0	34.3	43.1	23.9	12.8	18.6	0.0	18.6		1.6	0.0	72	Fragmented littoral forest	Mandena, Madagascar	Eppley et al. [45]
*Prolemur simus*	24	–	–	–	–	98	–	–	0.5	–	0.5	–	1.5	95^c^	7	Submontane rain forest	Ranomafana, Madagascar	Tan [26]
*Macaca assamensis*	12	75.5	1.8	–	0.0	77.3	–	1.3	20.1	0.1	20.2	–	1.3	71.2^d^	78	Limestone seasonal rain forest	Nonggang, China	Huang et al. [96]
*M. assamensis*	12	74.1	3.3	–	0.0	77.4	–	2.7	17.4	–	17.4	–	2.5	48.7^d^	69	Limestone seasonal rain forest	Nonggang, China	Zhou et al. [68]

*YL* young leaves, *ML* mature leaves, *SH* shoots, *GRB* grass blades, *TL* total leaves, *ST* stems, *PT* petioles, *FL* flowers, *FR* fruits, *S* seeds, *TF* total fruits, *AP* animal prey, *OS* other

^a^From Table 1 in Twinomugisha et al. [100], we took the average of the values for groups G and N during Time 1 (January–September 1998) and then averaged that value with the value for Time 2 (January–August 2003)

^b^ *Arundinaria alpina*

^c^ *Cathariostachys madagascariensis*

^d^ *Several bamboo species*

Giant and red pandas are arguably the best known obligate specialist folivores, exploiting diets consisting almost entirely of bamboo [34, 94]. Neither species exhibits an ability to cope with intensive habitat degradation [34, 94]. Among primates, some bamboo lemurs appear to be the most inclined towards extreme specialization [26]. In particular, the greater bamboo lemur (*Prolemur simus*) consumes a diet of 95% bamboo [26] and does not appear to exist outside of bamboo forest habitat [101, 102]. *P. simus* also relies heavily on an unusually cyanogenic bamboo species [95] and is probably the only ‘obligate specialist’ on bamboo among the bamboo-eating primates. Indeed, recent studies of several other bamboo lemurs (*Hapalemur* spp.) found they can survive in habitats without bamboo, consuming more species-rich diets in these habitats, including a high percentage of graminoids in the cases of *H. alaotrensis* [103] and *H. meridionalis* [36]. The increased consumption of graminoids by these *Hapalemur* spp. provides an interesting parallel to the Bale monkeys in our study, which also consumed more graminoids at fragmented sites where bamboo is scarce. Overall, it appears that, with the exception of *Prolemur simus*, bamboo eating primates are more dietarily flexible than giant and red pandas. This pattern is consistent with the evidence that the bamboo feeding adaptation in pandas is much older than it is for any of the bamboo feeding primates (e.g., [69, 93–95]).

### Implications for conservation and management

Our study revealed that, like most other bamboo-eating primates, Bale monkeys have the flexibility to cope with changes in the identity and abundance of foods resulting from habitat degradation and loss of bamboo, at least over the short-term. More intensive long-term studies of Bale monkeys in both fragmented and continuous habitats are, however, needed to examine and address some of the potential drawbacks of life in fragments. The greatest conservation concern raised by our study is that of human-monkey conflict at fragmented sites, especially at Patchy fragment. As in many other sites where primates crop raid [104], humans near fragments in our study sometimes responded to Bale monkey crop raiding in a manner that put Bale monkeys at risk, hunting them with spears and dogs. A more detailed study of this human-monkey conflict and its impact on Bale monkey survivorship in fragments should be a priority along with developing and implementing strategies to mitigate this conflict [105]. Any Bale monkey habitat restoration programs undertaken at fragments should focus on increasing fragment sizes, minimizing edge effects, incorporating matrix habitats into management plans, and mitigating human monkey-conflict (cf., [88, 106]). Moreover, the remaining continuous bamboo forest habitat in the southern Ethiopian Highlands should be protected from further deforestation both to best ensure the long-term persistence of Bale monkeys [93] and to prevent the functional homogenization of biodiversity in this important region for conservation [19, 107].

## Conclusions

Bale monkeys in fragments have smaller group sizes, and experience lower food availability and habitat quality relative to those in continuous forest ([66]; This study). Consequently, they consume more diverse species-rich diets, including more secondary and cultivated food resources. While Bale monkeys are the only specialized members of a genus, *Chlorocebus*, whose other five species are all ecological generalists, we hypothesize that they have either retained the ancestral *Chlorocebus* ability to fall back on a generalist diet where necessary or that populations in fragments have reacquired this ability through interbreeding with parapatric grivet (*C. aethiops*) or vervet (*C. pygerythrus*) populations. Despite the encouraging dietary flexibility documented among Bale monkeys in our study, the long-term conservation prospects for populations in forest fragments remain unclear and will require long-term population monitoring and conservation actions to ensure their persistence in the southern Ethiopian Highlands.

## Additional files

**Additional file 1.** Stem density of all plant species (≥ 2 m tall) within vegetation quadrats in the home ranges of study groups. Continuous A (n = 9110 stems), Continuous B (n = 5410 stems), Patchy (n = 3388 stems) and Hilltop (n = 2312) groups (* exotic species).

**Additional file 2.** Sample based rarefaction curves of plant species consumed by Bale monkeys among four study groups. Samples were collected in the continuous forest (Continuous A, N = 52 days (A); Continuous B, N = 54 days (B) and forest fragments (Patchy fragment, N = 62 days (C); Hilltop fragment, N = 67 days (D). The red (rarefaction) curves represent the cumulative number of plant species consumed by the study groups and blue curves represent the 95% confidence intervals.

**Additional file 3.** The proportion of feeding records devoted to consuming different plant growth forms by the four study groups. Proportions were summarized from N = 12 months, mean ± SE.

## Abbreviations

CEES: Centre for Ecological and Evolutionary Synthesis
IUCN: International Union for Conservation of Nature
DBH: diameter at breast height
BA: basal area
*H’*: Shannon–Wiener index
*D*: dominance index
*J*: evenness index
C_H_: Morisita–Horn’s similarity index
PAST: PAleontological Statistics
ANOVA: analysis of variance
Tukey HSD: Tukey honest significant difference test
GLM: generalized linear model
SE: standard error
vs.: versus
Cont_A: Continuous A
Cont_B: Continuous B
IQD: interquartile distance
PSMs: plant secondary metabolites
BYL: bamboo young leaves
NBYL: non-bamboo young leaves
BSH: bamboo shoots
FL: flowers
FR: fruit
ST: stems
PT: petioles
S: seeds
IN: insects
OS: others
OYL: young leaves except bamboo and grass
ML: mature leaves
BSH: bamboo shoots
GRL: grass blades
TL: total leaves
TF: total Fruits
GU: gum
AP: animal prey
CF: continuous forest
FF: fragmented forest
sp.: species
