# Supplementary material for: Dietary flexibility of Bale monkeys (Chlorocebus djamdjamensis) in southern Ethiopia: effects of habitat degradation and life in fragments
Source: BMC Ecol. 2018 Feb 6;18:4. doi: 10.1186/s12898-018-0161-4 (PMC5801891; doi:10.1186/s12898-018-0161-4)
Supplement: Supplementary file 1 — Additional file 1. Stem density of all plant species (≥ 2 m tall) within vegetation quadrats in the home ranges of study groups. Continuous A (n = 9110 stems), Continuous B (n = 5410 stems), Patchy (n = 3388 stems) and Hilltop (n = 2312) groups (* exotic species). [file 12898_2018_161_MOESM1_ESM.docx]

**Additional files**

Additional file 1. Stem density of all plant species (≥ 2 m tall) within vegetation quadrats in the home ranges of study groups. Continuous A (n=9110 stems), Continuous B (n=5410 stems), Patchy (n=3388 stems) and Hilltop (n=2312) groups (** exotic species*)

| **Continuous forest** | | | | | | | | | |
| --- | --- | --- | --- | --- | --- | --- | --- | --- | --- |
|  |  |  |  | Continuous A | | Continuous B | | Continuous Combined | |
| No. | Species | Family | Type | No. of stem/ha | % of Stem density | No. of stem /ha | % of stem density | Mean No. of stem/ha | Av. % of stem density |
| 1 | *Arundinaria alpina* | Poaceae | Bamboo | 7720.00 | 84.74 | 4713.33 | 87.12 | 6216.67 | 85.93 |
| 2 | *Galiniera saxifraga* | Rubiaceae | Tree | 311.67 | 3.42 | 163.33 | 3.02 | 237.50 | 3.22 |
| 3 | *Mikaniopsis clematoides* | Asteraceae | Liana | 306.67 | 3.37 | 26.67 | 0.49 | 166.67 | 1.93 |
| 4 | *Urera hypselodenron* | Urticaceae | Liana | 166.67 | 1.83 | 108.33 | 2.00 | 137.50 | 1.92 |
| 5 | *Cyphostemma adenocaule* | Vitaceae | Liana | 180.00 | 1.98 | 76.67 | 1.42 | 128.33 | 1.70 |
| 6 | *Acanthopale pubescens* | Acanthaceae | Herb | 26.67 | 0.29 | 93.33 | 1.73 | 60.00 | 1.01 |
| 7 | *Rubus apetalus* | Rosaceae | Shrub | 93.33 | 1.03 | 36.67 | 0.68 | 65.00 | 0.85 |
| 8 | *Dombeya torrida* | Sterculiaceae | Tree | 80.00 | 0.88 | 20.00 | 0.37 | 50.00 | 0.62 |
| 9 | *Oxystelma bornouense* | Asclepiadaceae | Liana | 26.67 | 0.29 | 50.00 | 0.92 | 38.33 | 0.61 |
| 10 | *Brucea antidysenterica* | Simarubaceae | Tree | 11.67 | 0.13 | 53.33 | 0.99 | 32.50 | 0.56 |
| 11 | *Crotalaria rosenii* | Fabaceae | Shrub | 53.33 | 0.59 | 0.00 | 0.00 | 26.67 | 0.29 |
| 12 | *Discopodium penninervium* | Solanaceae | Tree | 10.00 | 0.11 | 20.00 | 0.37 | 15.00 | 0.24 |
| 13 | *Schifflera volkensii* | Araliaceae | Tree | 31.67 | 0.35 | 6.67 | 0.12 | 19.17 | 0.24 |
| 14 | *Myrsine melanophloeos* | Myrsinaceae | Tree | 20.00 | 0.22 | 13.33 | 0.25 | 16.67 | 0.23 |
| 15 | *Jasminum abyssinicum* | Oleaceae | Liana | 16.67 | 0.18 | 6.67 | 0.12 | 11.67 | 0.15 |
| 16 | *Bersama abyssinica* | Melianthaceae | Tree | 10.00 | 0.11 | 8.33 | 0.15 | 9.17 | 0.13 |
| 17 | *Hagenia abyssinica* | Rosaceae | Tree | 16.67 | 0.18 | 3.33 | 0.06 | 10.00 | 0.12 |
| 18 | *Leggera sp.* | Asteraceae | Tree | 6.67 | 0.07 | 3.33 | 0.06 | 5.00 | 0.07 |
| 19 | *Embelia schimperi* | Embenaceae | Liana | 3.33 | 0.04 | 3.33 | 0.06 | 3.33 | 0.05 |
| 20 | *Zehneria scabra* | Cucurbitaceae | Liana | 3.33 | 0.04 | 3.33 | 0.06 | 3.33 | 0.05 |
| 21 | *Hypericum revoltum* | Hypericeae | Tree | 8.33 | 0.09 | 0.00 | 0.00 | 4.17 | 0.05 |
| 22 | *Maytenus obscura* | Celastraceae | Tree | 3.33 | 0.04 | 0.00 | 0.00 | 1.67 | 0.02 |
| 23 | *Allophylus macrobothys* | Sapindaceae | Tree | 3.33 | 0.04 | 0.00 | 0.00 | 1.67 | 0.02 |
| **Forest fragment** | | | | | | | | | |
|  |  |  |  | Patchy | | Hilltop | | Fragment Combined | |
| No. | Species | Family | Type | No. of stem/ha | % of stem density | No. of stem/ha | % of stem density | Mean No. of stem/ha | Av. % of stem density |
| 1 | *Arundinaria alpina* | Poaceae | Bamboo | 1341.43 | 39.59 | 37.14 | 1.61 | 689.29 | 20.60 |
| 2 | *Rubus apetalus* | Rosaceae | Shrub | 517.14 | 15.26 | 450.00 | 19.46 | 483.57 | 17.36 |
| 3 | *Bothriocline schimperi* | Asteraceae | Shrub | 282.86 | 8.35 | 347.14 | 15.01 | 315.00 | 11.68 |
| 4 | *Myrsine melanophloeos* | Myrsinaceae | Tree | 397.14 | 11.72 | 85.71 | 3.71 | 241.43 | 7.71 |
| 5 | *Galiniera saxifraga* | Rubiaceae | Tree | 66.93 | 1.98 | 101.43 | 4.39 | 84.18 | 3.18 |
| 6 | *Ilex mitis* | Aquifoliaceae | Tree | 21.80 | 0.64 | 108.81 | 4.71 | 65.30 | 2.67 |
| 7 | *Discopodium penninervium* | Solanaceae | Tree | 117.88 | 3.48 | 38.57 | 1.67 | 78.23 | 2.57 |
| 8 | *Maesa lanceolata* | Myrsinaceae | Tree | 153.12 | 4.52 | 10.24 | 0.44 | 81.68 | 2.48 |
| 9 | *Persicaria nepalensis* | Caryophyllaceae | Herb | - | - | 108.57 | 4.70 | 54.29 | 2.35 |
| 10 | *Urera hypselodenron* | Urticaceae | Liana | 47.14 | 1.39 | 75.71 | 3.27 | 61.43 | 2.33 |
| 11 | *Mikaniopsis clematoides* | Asteraceae | Liana | 44.29 | 1.31 | 72.86 | 3.15 | 58.57 | 2.23 |
| 12 | *Jasminum abyssinicum* | Oleaceae | Liana | 47.14 | 1.39 | 65.71 | 2.84 | 56.43 | 2.12 |
| 13 | *Eucalyptus globulus** | Myrtaceae | Tree | - | - | 89.29 | 3.86 | 44.64 | 1.93 |
| 14 | *Canthium oligocarpum* | Rubiaceae | Tree | 50.26 | 1.48 | 51.43 | 2.22 | 50.85 | 1.85 |
| 15 | *Bersama abyssinica* | Melianthaceae | Tree | 60.74 | 1.79 | 29.40 | 1.27 | 45.07 | 1.53 |
| 16 | *Senecio sp.* | Asteraceae | Shrub | - | - | 65.71 | 2.84 | 32.86 | 1.42 |
| 17 | *Vangueria madagascariensis* | Rubiaceae | Liana | 35.71 | 1.05 | 40.00 | 1.73 | 37.86 | 1.39 |
| 18 | *Brucea antidysenterica* | Simarubaceae | Tree | 28.57 | 0.84 | 41.43 | 1.79 | 35.00 | 1.32 |
| 19 | *Pteridium aquilinum* | Dennstaedtiaceae | Fern | - | - | 47.14 | 2.04 | 23.57 | 1.02 |
| 20 | *Jasminum stans* | Oleaceae | Liana | - | - | 42.86 | 1.85 | 21.43 | 0.93 |
| 21 | *Achyranthes aspera* | Amaranthaceae | Herb | - | - | 37.14 | 1.61 | 18.57 | 0.80 |
| 22 | *Solanecio gigas* | Asteraceae | Shrub | - | - | 37.14 | 1.61 | 18.57 | 0.80 |
| 23 | *Vernonia rueppellii* | Asteraceae | Shrub | 28.57 | 0.84 | 17.14 | 0.74 | 22.86 | 0.79 |
| 24 | *Hypericum revoltum* | Hypericeae | Tree | 5.03 | 0.15 | 33.10 | 1.43 | 19.06 | 0.79 |
| 25 | *Oxystelma bornouense* | Asclepiadaceae | Liana | 4.29 | 0.13 | 31.43 | 1.36 | 17.86 | 0.74 |
| 26 | *Pycnostachys eminii* | Lamiaceae | Shrub | 32.86 | 0.97 | 11.43 | 0.49 | 22.14 | 0.73 |
| 27 | *Maytenus obscura* | Celastraceae | Tree | 11.53 | 0.34 | 18.57 | 0.80 | 15.05 | 0.57 |
| 28 | *Cyphostemma adenocaule* | Vitaceae | Liana | - | - | 25.71 | 1.11 | 12.86 | 0.56 |
| 29 | *Lobelia giberroa* | Lobeleiaceae | Shrub | - | - | 22.86 | 0.99 | 11.43 | 0.49 |
| 30 | *Hagenia abyssinica* | Rosaceae | Tree | 5.77 | 0.17 | 18.81 | 0.81 | 12.29 | 0.49 |
| 31 | *Vernonia sp.* | Asteraceae | Shrub | - | - | 20.83 | 0.90 | 10.42 | 0.45 |
| 32 | *Clematis hirsuta* | Ranunculaceae | Liana | 15.71 | 0.46 | 10.00 | 0.43 | 12.86 | 0.45 |
| 33 | *Urtica simensis* | Urticaceae | Herb | - | - | 20.00 | 0.87 | 10.00 | 0.43 |
| 34 | *Phyllanthus leucanthus* | Euphorbiaceae | Shrub | - | - | 15.71 | 0.68 | 7.86 | 0.34 |
| 35 | *Ritchiea albersii* | Capparaceae | Tree | - | - | 15.71 | 0.68 | 7.86 | 0.34 |
| 36 | *Cupressus lusitanica** | Cupressaceae | Tree | 14.29 | 0.42 | 5.12 | 0.22 | 9.70 | 0.32 |
| 37 | *Schefflera volkensii* | Araliaceae | Tree | 8.04 | 0.24 | 9.05 | 0.39 | 8.55 | 0.32 |
| 38 | *Rubus volkensii* | Rosaceae | Shrub |  |  | 11.43 | 0.49 | 5.71 | 0.25 |
| 39 | *Thalictrum rhynchocarpum* | Ranunculaceae | Herb | - | - | 11.43 | 0.49 | 5.71 | 0.25 |
| 40 | *Vernonia myriantha* | Asteraceae | Shrub | - | - | 11.43 | 0.49 | 5.71 | 0.25 |
| 41 | *Embelia schimperi* | Embenaceae | Liana | 14.29 | 0.42 | - | - | 7.86 | 0.23 |
| 42 | *Nuxia congesta* | Loganiaceae | Tree | 11.53 | 0.34 | - | - | 5.77 | 0.17 |
| 43 | *Juniperus procera* | Cupressaceae | Tree | 1.48 | 0.04 | 3.69 | 0.16 | 2.59 | 0.10 |
| 44 | *Lagenaria abyssinica* | Cucurbitaceae | Liana | - | - | 4.29 | 0.19 | 2.14 | 0.09 |
| 45 | *Schefflera abyssinica* | Araliaceae | Tree | 5.03 | 0.15 |  |  | 2.51 | 0.07 |
| 46 | *Prunus africana* | Rosaceae | Tree | 1.43 | 0.04 | 2.26 | 0.10 | 1.85 | 0.07 |
| 47 | *Lactuca glandulifera* | Compositae | Liana | 4.29 | 0.13 | - | - | 2.14 | 0.06 |
| 48 | *Solanecio mannii* | Asteraceae | Shrub | 4.29 | 0.13 | - | - | 2.14 | 0.06 |
| 49 | *Maytenus spp* | Celastraceae | Shrub | - | - | 2.86 | 0.12 | 1.43 | 0.06 |
| 50 | *Unidentified* | Unidentified | Shrub | - | - | 2.86 | 0.12 | 1.43 | 0.06 |
| 51 | *Croton macrostachyus* | Euphorbiaceae | Tree | 2.86 | 0.08 | - | - | 1.43 | 0.04 |
| 52 | *Allophyllus abyssinicus* | Sapindaceae | Tree | 2.17 | 0.06 | - | - | 1.08 | 0.03 |
| 53 | *Erythrina brucei* | Papilionaceae | Tree | 1.43 | 0.04 | 1.43 | 0.06 | 0.71 | 0.03 |
| 54 | *Euphorbia abyssinica* | Euphorbiacea | Tree | - | - | 1.43 | 0.06 | 0.71 | 0.03 |
| 55 | *Acacia mearansii** | Fabaceae | Tree | 1.43 | 0.04 | 0.00 | 0.00 | 0.71 | 0.02 |
