# Supplementary material for: Dietary flexibility of Bale monkeys (Chlorocebus djamdjamensis) in southern Ethiopia: effects of habitat degradation and life in fragments
Source: BMC Ecol. 2018 Feb 6;18:4. doi: 10.1186/s12898-018-0161-4 (PMC5801891; doi:10.1186/s12898-018-0161-4)
Supplement: Supplementary file 2 — Additional file 2. Sample based rarefaction curves of plant species consumed by Bale monkeys among four study groups. Samples were collected in the continuous forest (Continuous A, N = 52 days (A); Continuous B, N = 54 days (B) and forest fragments (Patchy fragment, N = 62 days (C); Hilltop fragment, N = 67 days (D). The red (rarefaction) curves represent the cumulative number of plant species consumed by the study groups and blue curves represent the 95% confidence intervals. [file 12898_2018_161_MOESM2_ESM.docx]

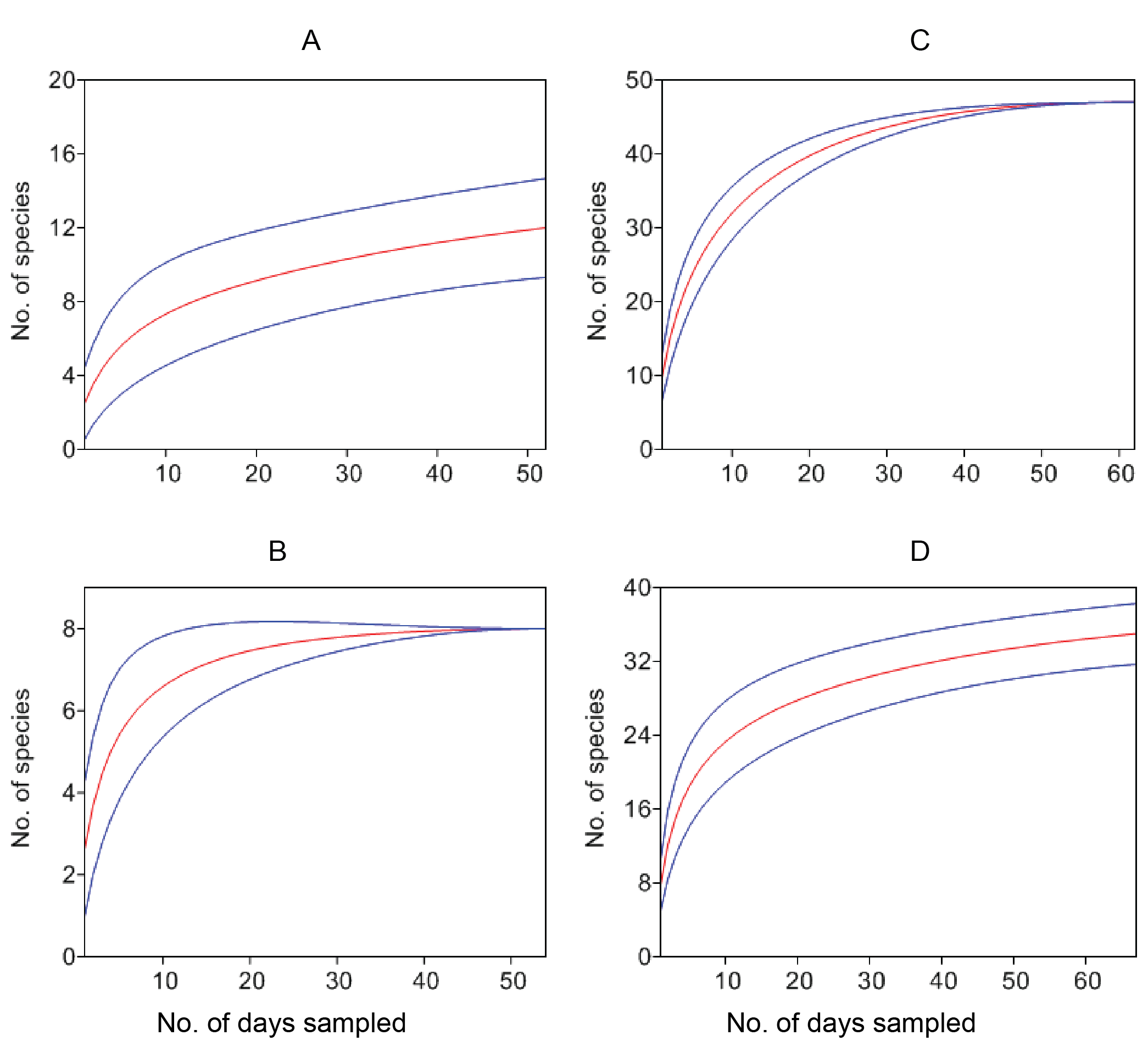


Additional file 2. Sample based rarefaction curves of plant species consumed by Bale monkeys among four study groups. Samples were collected in the continuous forest (Continuous A, N = 52 days (A); Continuous B, N = 54 days (B) and forest fragments (Patchy fragment, N = 62 days (C); Hilltop fragment, N = 67 days (D). The red (rarefaction) curves represent the cumulative number of plant species consumed by the study groups and blue curves represent the 95% confidence intervals.
