# Supplementary material for: Dietary flexibility of Bale monkeys (Chlorocebus djamdjamensis) in southern Ethiopia: effects of habitat degradation and life in fragments
Source: BMC Ecol. 2018 Feb 6;18:4. doi: 10.1186/s12898-018-0161-4 (PMC5801891; doi:10.1186/s12898-018-0161-4)
Supplement: Supplementary file 3 — Additional file 3. The proportion of feeding records devoted to consuming different plant growth forms by the four study groups. Proportions were summarized from N = 12 months, mean ± SE. [file 12898_2018_161_MOESM3_ESM.docx]

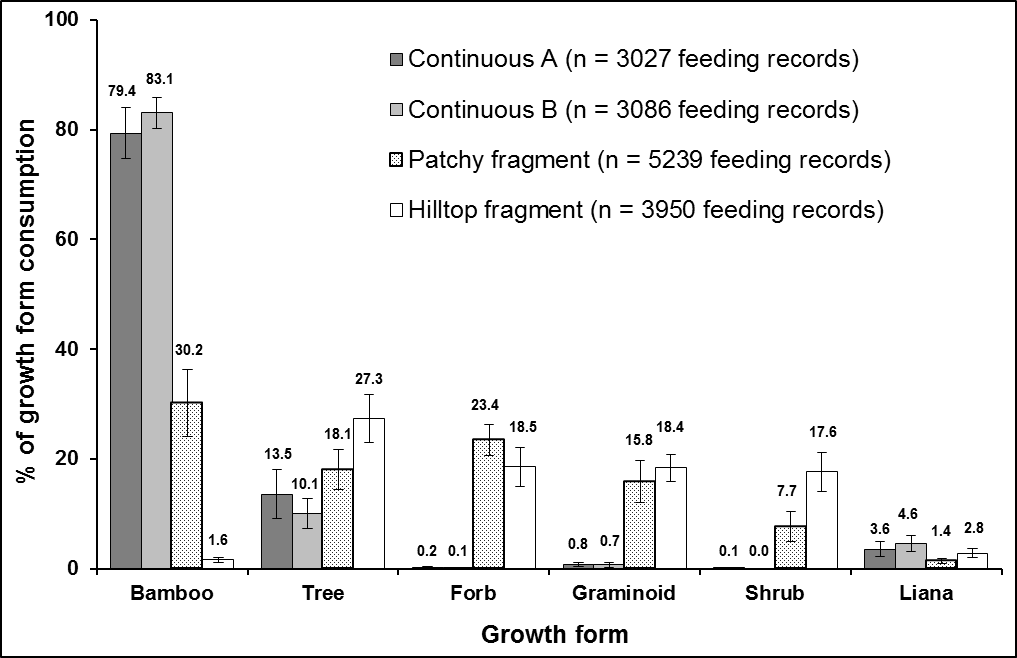


Additional file 3. The proportion of feeding records devoted to different plant growth forms by the four study groups. Proportions were summarized from N=12 months, mean±SE.
